# The effectiveness and safety of dual antiplatelet therapy in ischemic cerebrovascular disease with intracranial and extracranial arteriostenosis in Chinese patients: A randomized and controlled trail

**DOI:** 10.1097/MD.0000000000005497

**Published:** 2017-01-10

**Authors:** Feng-Tong Zuo, Hui Liu, Hui-Jun Wu, Na Su, Jie-Qiong Liu, Ai-Qin Dong

**Affiliations:** a Department of Neurology, Cangzhou Central Hospital; b Department of Internal Medicine, Cangzhou Peace Hospital, Cangzhou, Hebei, China.

**Keywords:** aspirin, clopidogrel sulfate, intracranial arteriostenosis, ischemic cerebrovascular disease, stroke

## Abstract

**Background:**

There are limited data on the effect of dual antiplatelet treatment with clopidogrel plus aspirin in patients with ischemic cerebrovascular disease and intracranial and extracranial arteriostenosis. The aim of our study was to evaluate the efficacy and safety of aspirin plus clopidogrel in the treatment of ischemic cerebrovascular disease with intracranial and extracranial arteriostenosis.

**Methods:**

Patients with clinically evident acute cerebral infarction or transient ischemic attack combined with intracranial and extracranial arteriostenosis (greater than 50%) who were unsuitable or reluctance to perform stent implantation were enrolled in this study. We randomly assigned these patients to receive clopidogrel (75 or 50 mg) plus aspirin (100 mg) or aspirin (100 mg) once daily through 90 days, and followed them for 90 days. We examined the main endpoints including the recurrence of stroke, death from cardiovascular causes, and bleeding events.

**Results:**

In all, 200 patients were recruited and followed for 90 days. Ischemic stroke occurred in 6 patients (9.1%) treated with 50 mg clopidogrel and aspirin, 6 patients (9.1%) receiving 75 mg clopidogrel and aspirin, whereas 19 patients (27.9%) in the aspirin group (aspirin alone vs copidogrel 50 mg plus aspirin; 95% confidence intervals 1.704–23.779, *P* < 0.05; aspirin alone vs copidogrel 75 mg plus aspirin; 95% confidence intervals 1.190–13.240, *P* < 0.05). There were more hemorrhagic events among recipients (3 patients [2.3%]) in the copidogrel plus aspirin group than aspirin recipients (0 patient [0%]), including 1 subcutaneous hemorrhage in the group of 50 mg clopidogrel and aspirin, doubling the number of nasal and gum bleeding in the group of 75 mg clopidogrel and aspirin (*P* > 0.05). No intracranial hemorrhage and gastro-intestinal hemorrhage occurred in these 3 groups.

**Conclusion:**

Accordingly, 50 mg clopidogrel plus aspirin, and 75 mg clopidogrel plus aspirin were all superior to aspirin alone as stroke prevention in patients with cerebral infarction or transient ischemic attack combined with intracranial and extracranial arteriostenosis. The effect of secondary stroke prevention was similar between 50 mg clopidogrel plus aspirin and 75 mg clopidogrel plus aspirin. The therapy of 75 mg clopidogrel plus aspirin resulted in a worrisome tread in bleeding events.

## 1 Introduction

Ischemic cerebrovascular disease (ICVD) is the main cause of disability and death among all adults.^[1]^ In China, there are approximately 3 million new strokes every year.^[2]^ Moreover, patients with stroke or transient ischemic attack (TIA) contribute to the increased risk of developing recurrent ICVD. Significantly, stenosis of cerebral vessels is a common cause of stroke in the world, and is related with a high risk of recurrent stroke.^[3,4]^ As documented, extracranial stenosis is more common in Caucasians, whereas intracranial stenosis is more common in Asians, Hispanics, and also African-Americans.^[5,6]^ Intracranial atherosclerosis results in 30% to 50% of strokes in Asia^[7]^ and 8% to 10% of strokes in North America.^[8]^ Frequently, surgical treatment (extracranial–intracranial bypass surgery) has been demonstrated to be the most commonly utilized for stroke prevention in patients with symptomatic intracranial atherosclerosis.^[9]^ However, available data have indicated a high complication rate after bypass procedure.^[10]^ Moreover, because of the indications and contraindications, surgical and interventional treatments are not performed for these patients. Thus, new and effective therapy is needed to treat this high-risk disease to prevent recurrent stroke.

Antiplatelet therapy has been exhibited to exert important roles in the secondary prevention of stroke.^[11]^ Significantly, low-dose aspirin is effective to reduce ischemic events in patients above a certain risk threshold.^[11]^ Nevertheless, in many cases, aspirin alone is not sufficient to aid the prevention of ischemic events in patients with high risk. Fortunately, clopidogrel versus aspirin in patients at risk of ischaemic events (CAPRIE) study has indicated that the effect of clopidogrel is superior to aspirin in the ischemic stroke patients, decreasing the relative risk for ischemic stroke by 8.7% versus aspirin (*P* = 0.043).^[12]^ Recently, a meta-analysis has been exhibited that dual antiplatelet therapy may safely and effectively decrease the recurrence of stroke and vascular outcomes in patients with TIA or ischemic stroke, compared with monotherapy.^[13]^ Collectively, a former study has demonstrated that clopidogrel plus aspirin reduces infarction in acute stroke or TIA patients with large artery stenosis, which further indicates the combination of aspirin and clopidogrel is more effective than aspirin alone in stroke patients with large artery disease.^[14]^ Another previous study has demonstrated that dual therapy with clopidogrel and aspirin is more effective than aspirin alone in reducing asymptomatic embolization in patients with carotid stenosis.^[15,16]^ However, in the Management and Avoidance trial, the combination of clopidogrel 75 mg plus aspirin has indicated no clear superiority for stroke prevention over aspirin alone,^[17]^ and the results are similar in patients with a lacunar stroke in the secondary prevention of small subcortical strokes trial.^[18]^ Because of these inconsistent data, we proposed to resolve this uncertainty. On the contrary, considerable differences exist between Chinese and Western population with respect to health conditions, genetic background, and medicine tradition. Remarkably, in China, the incidence of cerebral bleeding induced by antiplatelet drugs is high in the secondary prevention of stroke.^[19]^ More importantly, dual antiplatelet treatment with clopidogrel plus aspirin is often used to treat patients with purely intracranial arteriostenosis, but there are limited data on the effect of clopidogrel plus aspirin in a broad population of ICVD patients with intracranial and extracranial arteriostenosis. Moreover, the combination of aspirin and clopidogrel has not yet proven effective and safe in the secondary prevention of stroke and TIA.^[20,21]^ Significantly, Asano et al^[22]^ have indicated that lower maintenance dose of clopidogrel (50 mg) is appropriate for Japanese patients. Thus, low-dose (50 mg) clopidogrel may be acceptable for Chinese patients. Thus, a study to compare safety and efficacy of 50 mg with 75 mg clopidogrel is required. To our knowledge, there were limited studies comparing the efficacy and safety between 50 and 75 mg clopidogrel in Chinese patients with ICVD, and intracranial and extracranial arteriostenosis.

Hence, we carried out the trial to verify the hypothesis that the combination of clopidogrel and aspirin may have a greater benefit in prevention of recurrent stroke in patients from China who had ICVD and intracranial and extracranial arteriostenosis. In addition, we compare the efficacy of secondary stroke prevention between 50 and 75 mg clopidogrel. Moreover, the potential bleedings were estimated after adding aspirin to clopidogrel. Our study will shed light on the theoretical basis for the application of the combination of clopidogrel and aspirin in the secondary prevention of stroke in Chinese subjects with intracranial and extracranial arteriostenosis.

## 2 Materials and methods

### 2.1 Study design

This research was a prospective, randomized, single-center study of efficacy of clopidogrel plus aspirin as compared with aspirin alone in patients with clinically evident ICVD combined with intracranial and extracranial arteriostenosis. The trial proposal was approved by the ethics committee of the hospital. All enrolled patients or family members signed written informed consent.

### 2.2 Participants

Between January 2013 and March 2014, all patients with possible clinical ischemic cerebrovascular events during the period of hospitalization were subjected to computed tomography or magnetic resonance imaging of the head and neck. Patients were eligible when they met the following inclusive criteria: patients with age ranging from 45 to 80 years; diagnosis of an acute cerebral infarction (CI) or TIA; greater than 50% stenosis of internal carotid artery (ICA), middle cerebral artery (MCA), vertebral artery (VA), basilar artery (BA), and posterior cerebral artery (PCA) alone or in combination; unsuitable or reluctance to perform stent implantation.

Exclusion criteria were as follows: large-area CI or hemorrhagic CI; cardiogenic brain embolism; current peptic ulceration or history of systemic bleeding; platelet number less than 10^5^/mm^3^ or coagulopathy; systemic disease, for example, terminal malignancy or serious renal or liver disease; known contraindication to clopidogrel or aspirin; CI caused by rheumatoid disease or arterial inflammation; major surgery or trauma in the previous 3 months, or planning operation in the near future; gastrointestinal disorders or inability to obtain oral drugs; discontinuation of the study drug before testing.

Significantly, the characteristics of age, sex, smoking history, and also other vascular risk factors including diabetes, hyperlipidemia, hypertension, TIA, coronary heart disease, or cerebral infarction were assessed at baseline.

### 2.3 Procedures

Patients meeting the inclusion criteria were randomly assigned (1:1:1) to receive aspirin alone (100 mg), or clopidogrel (50 mg) plus aspirin (100 mg), or clopidogrel (75 mg) plus aspirin (100 mg). Patients in the aspirin alone group received a loading dose of 100 mg/d aspirin on day 1 through 90 days. Patients in clopidogrel (50 mg) plus aspirin (100 mg) group received clopidogrel at a dose of 50 mg/d and aspirin in a dose of 100 mg daily on day 1 through 90 days. Cases in clopidogrel (75 mg) plus aspirin (100 mg) group received clopidogrel at a dose of 75 mg/d and aspirin at a dose of 100 mg/d on day 1 through 90 days. Moreover, all patients were treated with standard therapy as appropriate (eg, hypoglycemic or antihypertensive drugs) at the discretion of the investigator and clinician. The appropriate standard therapy was emphasized to the investigator or clinician, who was offered with international guidelines.

We required all patients to return at 90 days for a consultation with a trained trial nurse or physician. If it was not feasible, the follow-up evaluations were acquired by telephone contact with the patient or the family doctor or caregiver. At these contacts, the occurrence of possible outcome events, changes in trial medication, and adverse events was recorded.

### 2.4 Endpoints

The main study endpoints were recurrence of ischemic stroke, death from any causes, and death from cardiovascular causes (including hemorrhage) in the first 90 days after CI or TIA. Stroke recurrence was determined as additional neurological deficit, and also corresponding positive lesions on diffusion-weighted imaging. All patients with the main study endpoints undertook brain CT scan to exclude intracranial hemorrhage.

Several hemorrhagic events were monitored in consideration of safety concerns based on the Global Utilization of Streptokinase and Tissue Plasminogen Activator for Occluded Coronary Arteries (GUSTO) definition, which includes intracranial hemorrhage and gastrointestinal bleeding. Moreover, nasal and gum bleeding were also examined.

### 2.5 Statistical analysis

All data were analyzed by means of SPSS 19.0 (SPSS Inc., Chicago, IL) and were tabulated using Microsoft software (Excel, Microsoft, Redmond, WA). All data were shown as the mean ± standard deviation (SD). Differences in baseline factors (sex, age, clinical characteristics, concomitant medications, risk factors) among groups were compared using the chi-square test. The treatment effects were compared with analysis of variance, and 95% confidence intervals (CIs). Statistical analysis of safety data was done with Pearson chi-square test. A *P* value less than 0.05 was regarded significant difference.

## 3 Results

### 3.1 Characteristics of the patients

**Figure 1:**
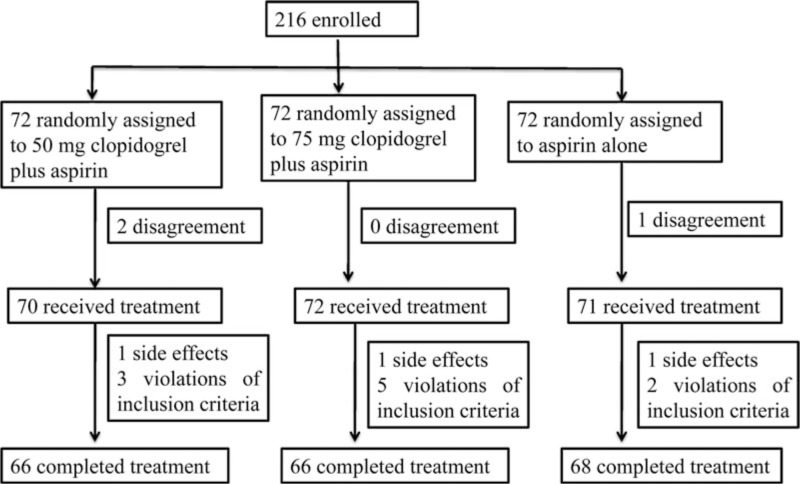
Profile of the clinical trial.

Between January 2013 and March 2014, a total of 216 patients with ICVD (CI or TIA, within 7 days of onset) plus intracranial and extracranial arteriostenosis were eligible and enrolled in our study. However, 16 of these patients were gradually excluded from the trial as they refused to provide the written informed consent, or had side effects or met at least 1 exclusion standard (Fig. 1). Thus, a total of 200 eligible subjects were included in our study. Among these patients, 66 received clopidogrel 50 mg plus aspirin, another 66 participants were treated using clopidogrel 75 mg plus aspirin, and the remaining 68 patients were treated using aspirin alone. Of note, these 200 patients completed the treatment.

The patients’ baseline characteristics in the study are shown in Table 1. In all 200 patients, the median age was 62 years, and 79 (39.5%) patients were female. About one-half patients had a TIA. There was no difference of baseline characteristics among these groups. There were no significant differences in age, sex, and disease category. The most prevalent risk factors in these 3 groups were smoking (clopidogrel 50 mg plus aspirin: 50%; clopidogrel 75 mg plus aspirin: 54.5%; aspirin: 48.5%), hypertension (clopidogrel 50 mg plus aspirin: 68.2%; clopidogrel 75 mg plus aspirin: 60.6%; aspirin: 65.2%), and hypercholesterolemia (clopidogrel 50 mg plus aspirin: 65.2%; clopidogrel 75 mg plus aspirin: 62.1%; aspirin: 58.8%). But there was also no significant difference in these risk features.

**Table 1:**
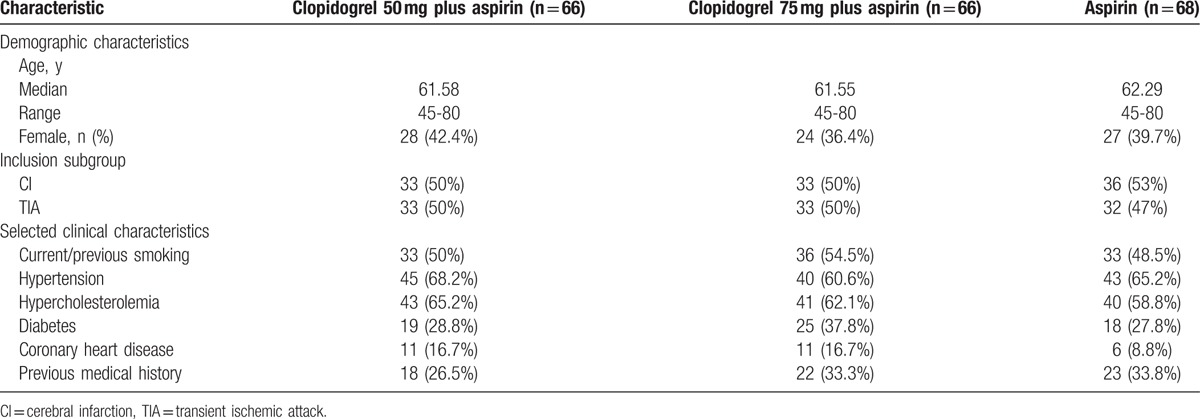
Baseline characteristics.

Medications taken by the patients were exhibited in Table 2. These figures depicted the maximal frequency of drug use at any time during the trial (evaluated at baseline and during the period of follow-up). All of the patients took aspirin and the study drug. Almost all of the patients took statin, and more than half used beta-blocker. Nearly one-half patients took diuretics and angiotensin-converting-enzyme inhibitors, and a quarter took angiotensin II receptor blockers.

**Table 2:**
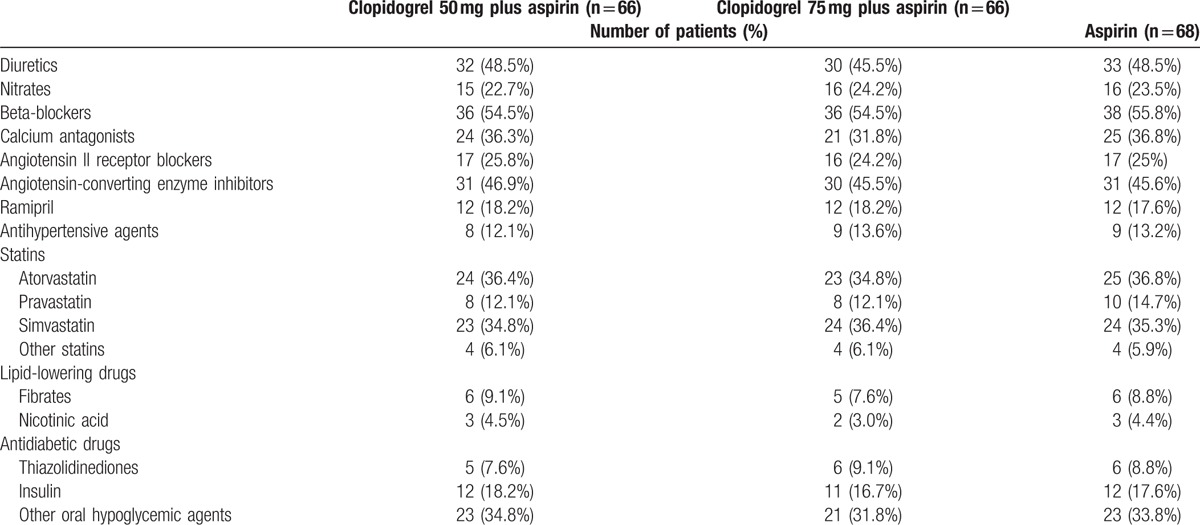
Concomitant medications.

### 3.2 Efficacy endpoints

During the period of follow-up, the ischemic stroke occurred in 6 patients (9.1%) treated with 50 mg clopidogrel and aspirin, 6 patients (9.1%) receiving 75 mg clopidogrel and aspirin, whereas 19 patients (27.9%) in the aspirin group (aspirin alone vs copidogrel 50 mg plus aspirin [95% CIs 1.704–23.779]; aspirin alone vs copidogrel 75 mg plus aspirin [95% CIs 1.190–13.240]) (Table 3). Both dual antiplatelet therapeutic approaches (copidogrel 50 mg plus aspirin, and copidogrel 75 mg plus aspirin) showed an obvious decrease in the recurrence of ischemic stroke (*P* < 0.05), comparing with that of the patients treated by aspirin alone.

**Table 3:**
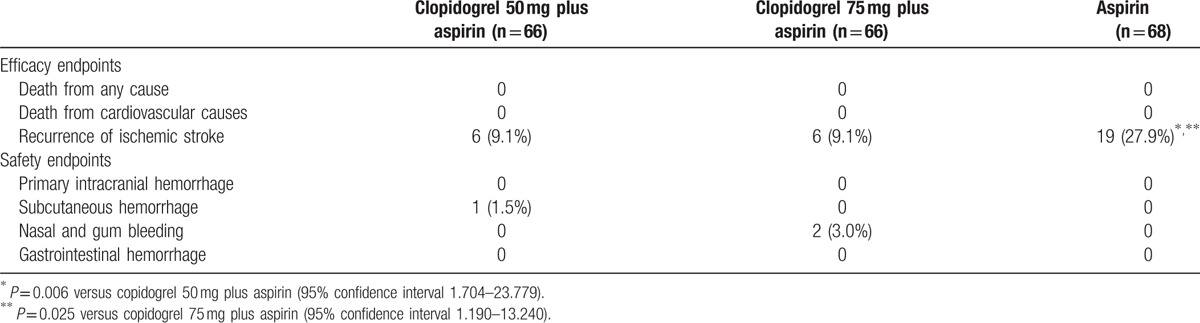
Efficacy and safety endpoints.

### 3.3 Safety endpoints

As shown in Table 3, hemorrhagic events occurred more frequently in the patients of clopidogrel and aspirin (3 patients [2.3%]) than in the recipients of aspirin alone (0 patient [0%]). However, no significant difference was observed in the risk of life-threatening bleedings. Moreover, there was no statistical significance in the increased incidence of hemorrhage between dual antiplatelet treatment group and aspirin group (*P* > 0.05). There was 1 case with subcutaneous hemorrhage in the group of 50 mg clopidogrel and aspirin, doubling the number of nasal and gum bleeding in the group of 75 mg clopidogrel and aspirin, but no difference was observed (*P* > 0.05). No primary intracranial hemorrhage and gastrointestinal hemorrhage occurred in these 3 groups.

## 4 Discussion

In Europe and United States, a 75 mg maintenance dose of clopidogrel is utilized. However, 75 mg clopidogrel can lead to higher platelet inhibition, which is a trade-off for higher bleeding complications. Significantly, Asano et al^[22]^ have indicated that lower maintenance dose of clopidogrel (50 mg) is appropriate for Japanese patients. Thus, low-dose (50 mg) clopidogrel may be acceptable for Chinese patients. Thus, a study to compare safety and efficacy of 50 mg with 75 mg clopidogrel is required. We consider that, even if efficacy and safety were similar between 50 and 75 mg clopidogrel, 50 mg clopidogrel would be useful because this drug is cost-effective. To our knowledge, there were limited studies comparing the efficacy and safety between 50 and 75 mg clopidogrel in Chinese patients with ICVD and intracranial and extracranial arteriostenosis. More significantly, we also compared the efficacy and evaluated the safety of combination of clopidogrel and aspirin over aspirin alone in reducing or preventing the recurrence of stroke in ICVD patients with intracranial and extracranial arteriostenosis. The results showed that early dual therapy with clopidogrel plus aspirin was more effective than aspirin alone in decreasing the presence of acute ischemic stroke. However, the dual therapy of clopidogrel plus aspirin resulted in a worrisome tread of increased bleeding events, compared with aspirin alone, but there was no difference. The efficiency of reducing stroke was similar between 50 mg clopidogrel plus aspirin group and 75 mg clopidogrel plus aspirin group. The risk of bleeding was lower in the group of 50 mg clopidogrel and aspirin, compared with 75 mg clopidogrel and aspirin, but no statistical difference was recorded. These results imply that clopidogrel plus aspirin is superior to aspirin alone in the early treatment of ICVD with intracranial and extracranial arteriostenosis.

As reported, aspirin is the first option of antithrombotic drug for the prevention of noncardioembolic stroke and TIA, but there is a relatively limited efficacy on recurrent events with a relative risk reduction of 13% to 22%.^[11,23]^ Excitedly, combining different antiplatelet medicines is thus a logical strategy to improve efficacy. Clopidogrel plus aspirin synergistically increased antagonism of platelet aggregation. The combination seemed to be well-tolerated and effective after intracranial stenting.^[24]^ For symptomatic patients with TIA or stroke because of a severe stenosis (70%–99%) of a major intracranial artery, Rahme et al^[25]^ reported a much lower risk of stroke with aspirin plus clopidogrel, when compared with the aspirin group in prior study provided by Kasner et al^[26]^ (16.3% vs 29%, on the basis of indirect comparison). However, the combination of aspirin and clopidogrel has not yet proven effective and safe in the secondary prevention of stroke and TIA.^[20,21]^

Except aspirin and clopidogrel, the patients in our study were treated with standard drugs, with frequent application of hypoglycemic, statins, beta-blockers, angiotensin-converting enzyme inhibitors, or antihypertensive drugs. The incidence of the efficacy endpoints was about 5.1% per month. In addition to this, we should take into consideration the balance between the reduction of stroke recurrence and the increasing risk of bleeding when using more reinforced antiplatelet treatment. In our analysis, there was 1 case with subcutaneous hemorrhage in the group of 50 mg clopidogrel and aspirin, doubling the number of nasal and gum bleeding in the group of 75 mg clopidogrel and aspirin. However, there was no statistical significance in hemorrhage in the group of 50 mg clopidogrel plus aspirin, and the group of 75 mg clopidogrel plus aspirin. Moreover, no significant difference was observed in the risk of life-threatening bleedings in these 3 groups. Despite this, a worrisome trend was noted that clopidogrel, especially 75 mg clopidogrel, was related with an increased rate of bleeding events. Unfortunately, another study has also shown that the fatal bleeding risk is higher in the group of 75 mg clopidogrel plus 75 mg aspirin versus monotherapy.^[20]^ Encouragingly, in 2013, Wang et al^[27]^ have indicated that the combination therapy with clopidogrel, and also aspirin (clopidogrel at a loading dosage of 300 mg on day 1, then 75 mg per day for 90 days, aspirin at a dosage of 75 mg daily on day 2 through 21 days) is better than aspirin alone for decreasing the stoke risk, and hemorrhage events are not increased. Another study has also indicated that loading with 300 mg clopidogrel added to 75 mg aspirin, followed by 75 mg clopidogrel and aspirin once daily to 90 days, reduces the incidence of recurrent stroke, without any severe bleeding.^[28]^ Accordingly, front-loading method is important in the secondary prevention of stroke. Although a worrisome trend was noted that clopidogrel, especially 75 mg clopidogrel, was related with an increase rate of bleeding events in our study, no difference was recorded between any 2 groups in the groups of 50 mg clopidogrel plus aspirin, 75 mg clopidogrel plus aspirin, and aspirin alone. Thus, the protocol of our study was feasible for secondary stroke prevention, but the sample size was small. In the future work, we will compare the effects of front-loading method and our protocol in larger multicenters with larger sample size to verify which method is more available in preventing stroke in patients with TIA or stroke combine with intracranial and extracranial arteriostenosis.

The incidence for stroke recurrence after TIA is up to 20% in the first 90 days, and most events accrue in the first few days.^[29]^ Thus, it is warranted to take emergency measures to prevent the occurrence of stroke. Wang et al^[30]^ have suggested that the combination therapy of clopidogrel and aspirin for patients with ischemic stroke or TIA within 7 days of onset is more effective than aspirin alone to reduce microembolic signals, without severe bleeding. The fast assessment of stroke and transientischaemic attack to prevent early recurrence (FASTER) pilot study also has suggested that the incidence of CI is 7.1% after treatment of clopidogrel plus aspirin once daily to 90 days, but 10.8% after treatment of aspirin alone once daily to 90 days, which exhibits a 3.7% risk benefit favoring clopidogrel and aspirin over aspirin alone.^[21]^ Consistent with these findings, our results indicate that combination therapy of clopidogrel and aspirin is superior to aspirin alone in reducing the recurrence of stroke for patients with CI or TIA within 7 days of onset. Accordingly, our findings and previous trials provide sufficient evidence to prefer the combination therapy of clopidogrel and aspirin over aspirin monotherapy as stroke prevention after ICVD. This might be explained by synergistic effects of 2 antiplatelet drugs due to different action mechanisms. Aspirin inhibits the aggregation of platelets through the cyclooxygenase pathway.^[31]^ Clopidogrel, an adenosine diphosphate glucose pyrophospheralase (ADP) receptor inhibitor, has to be metabolized in the liver to exert the antiaggregation functions. Collectively, ADP is 1 important mediator of hemostasis and thrombosis. Importantly, the metabolites of clopidogrel play crucial roles of reducing the risk of recurrent ischemic events by acting on the platelet P2Y12 receptor and suppressing the activation of ADP.^[32]^ It can be seen that clopidogrel and aspirin play important roles in the inhibition of platelet aggregation via different pathways, and the combined application of these 2 drugs has synergetic effects.

Although we obtained some significant evidences to confirm our hypothesis, there remained several drawbacks in the present analysis. The sample size was not large enough, which might weaken the statistical power. Furthermore, patients were recruited in 1 clinical center. Moreover, efficacy and safety of aspirin plus clopidogrel were obtained within a short follow-up period of 90 days. Because of these limitations, randomized trials in larger multicenters with larger sample size and longer time observation are warrant to identify the effectiveness and safety of combination of different drugs for second prevention of stroke in patients with TIA or stroke combine with intracranial and extracranial arteriostenosis. Last but not the least, our study did not use the front-loading method to measure the effect of clopidogrel and aspirin in avoiding the bleeding events; thus, we will do this work in future. Despite these drawbacks, to a certain degree, our results provided some preliminary evidence to prefer the combination regimen of clopidogrel sulfate plus aspirin over aspirin alone as stroke prevention in patients.

Taken together, our findings indicated that the use of 50 mg clopidogrel plus aspirin, and 75 mg clopidogrel plus aspirin was superior to aspirin alone in the aspect of reducing the incidence of ischemic stroke for patients with ICVD and intracranial and extracranial arteriostenosis. Moreover, the effect of secondary stroke prevention was similar between 50 mg clopidogrel plus aspirin group and 75 mg clopidogrel plus aspirin group. The dual therapy of 75 mg clopidogrel plus aspirin resulted in a worrisome tread in bleeding events.
